# Lumpectomy without radiation for ductal carcinoma in situ of the breast: 20-year results from the ECOG-ACRIN E5194 study

**DOI:** 10.1038/s41523-024-00622-w

**Published:** 2024-02-24

**Authors:** Jean L. Wright, Robert Gray, Habib Rahbar, Christopher E. Comstock, Judy A. Tjoe, Sunil Badve, Abram Recht, Joseph A. Sparano, Nancy E. Davidson, Antonio C. Wolff

**Affiliations:** 1 Department of Radiation Oncology, Johns Hopkins University School of Medicine, Baltimore, MD USA; 2 Dana Farber Cancer Institute, Boston, MA USA; 3 Fred Hutchinson Cancer Research Center, Seattle, WA USA; 4 Memorial Sloan Kettering Cancer Center, New York, NY USA; 5 Department of Surgical Oncology, Green Bay Oncology, Green Bay, WI USA; 6 Department of Pathology and Laboratory Medicine, Emory University School of Medicine, Emory University Winship Cancer Institute, Atlanta, GA USA; 7 Beth Israel Deaconess Medical Center, Harvard Medical School, Boston, MA USA; 8 Division of Hematology and Medical Oncology, Department of Medicine, Tisch Cancer Institute, Icahn School of Medicine at Mount Sinai, New York, NY USA; 9 Fred Hutchinson Cancer Center and University of Washington, Seattle, WA USA; 10 Johns Hopkins Women’s Malignancies Program, Johns Hopkins University School of Medicine, Baltimore, MD USA

**Keywords:** Breast cancer, Cancer prevention

## Abstract

We report the 20-year rate of ipsilateral breast event (IBE) for patients with ductal carcinoma in situ (DCIS) treated with lumpectomy without radiation on a non-randomized prospective clinical trial. Patients were enrolled in cohort 1: low- or intermediate-grade DCIS, size ≤ 2.5 cm (*n* = 561); or cohort 2: high-grade DCIS, size ≤ 1 cm (*n* = 104). The Kaplan–Meier method was used to estimate time-to-event distributions. Cox proportional hazard methods were used to estimate hazard ratios (HRs) and tests for significance for event times. 561 patients were enrolled in cohort 1 and 104 in cohort 2. After central pathology review, 26% in cohort 1 were recategorized as high-grade and 26% in cohort 2 as low- or intermediate-grade. Mean DCIS size was similar at 7.5 mm in cohort 1 and 7.8 mm in cohort 2. Surgical margin was ≥3 mm in 96% of patients, and about 30% received tamoxifen. Median follow-up was 19.2 years. There were 104 IBEs, of which 54 (52%) were invasive. The IBE and invasive IBE rates increased in both cohorts up to 15 years, then plateaued. The 20-year IBE rates were 17.8% for cohort 1 and 28.7% for cohort 2 (*p* = 0.005), respectively. Invasive IBE occurred in 9.8% and 15.1% (*p* = 0.09), respectively. On multivariable analysis, IBE risk increased with size and was higher in cohort 2, but grade and margin width were not significantly associated with IBE. For patients with DCIS treated with excision without radiation, the rate of IBE increased with size and assigned cohort mostly in the first 15 years.

## Introduction

Nearly 15 years after the 2009 National Institutes of Health (NIH) State of Science Conference recommended concerted efforts to decrease indolent ductal carcinoma in situ (DCIS) diagnoses unnecessary surgeries, and excessive adjuvant treatment^1^, the optimal management for patients with DCIS remains controversial^2^, with most patients undergoing lumpectomy followed by radiotherapy (RT) and/or endocrine therapy (ET). The long-term rate of ipsilateral breast events (IBE) after wide local excision (WLE) varies substantially with clinical, pathologic, and genomic factors^3–9^, and patient selection for omission of adjuvant therapy continues to be elusive. The Radiation Therapy Oncology Group (RTOG) trial 9804 is the largest modern randomized study evaluating the impact of RT omission in patients with “good risk” DCIS. The study defined low-risk DCIS based on clinical and pathologic criteria, including mammographic detection, size ≤ 2.5 cm, margins ≥ 3 mm, and low or intermediate nuclear grade, and randomized patients to RT vs observation with or without endocrine therapy. In this study, IBE rate with and without RT increased modestly but continuously over time up to 15 years, and there was a significant reduction in recurrence rate with RT even in this highly selected group of patients with lower risk DCIS (7.1% vs. 15.1%)^5^. The ECOG-ACRIN Cancer Research Group E5194 is a non-randomized, prospective study of observation after WLE without RT that enrolled patients in two cohorts of patients with study-defined low-risk clinical and pathologic characteristics: cohort 1 included patients with institutionally categorized low- or intermediate-grade DCIS spanning ≤ 2.5 cm, and cohort 2 included patients with high-grade DCIS spanning ≤ 1 cm. Like RTOG 9804, endocrine therapy was given at the provider’s discretion. We have previously reported 12-year outcomes of E5195, which showed an increasing rate of IBE with time, as well as a marked separation of IBE rate between the two cohorts^6^. In cohort 1, the 12-year IBE rate of 14.4% was similar to that in RTOG 9804, but in cohort 2, the 12-year IBE rate was significantly higher at 24.6%. It is important to recognize that the DICS grade was re-assigned after central pathology review to align with updated pathology reporting guidelines^10^ after all enrollment had been completed, as reported in the 12-year data^6^. As a result, 26% of patients in cohort 1 had pathology up-graded to high grade, and 26% of patients in cohort 2 had pathology down-graded to low- or intermediate grade. While the differences in IBE rate between the two cohorts reported in E5194 have been widely understood to relate specifically to DCIS grade, on multivariable analysis, centrally determined DCIS grade was not associated with IBE in that analysis^6^. At the same time, while size criteria for the two cohorts were different, the size of DCIS in enrolled patients in both cohorts was similar, and DCIS size was strongly associated with IBE. These factors leave unanswered questions about the relationship between clinical and pathological factors and the long-term risk of IBE. Importantly, at the 12- and 15-year timepoints, there did not appear to be a reduction in IBE rate in either E5194 or RTOG 9804. The present report provides updated results of E5194 with 20-year outcomes.

## Results

### Patient, DCIS, and treatment characteristics

Patient, DCIS, and treatment characteristics and follow-up are summarized in Table 1. While protocol-specified size limitations for DCIS were different between cohorts, mean size was actually similar at 7.5 mm in cohort 1 and 7.8 mm in cohort 2. DCIS grade is reported as the CAP grade defined on the central pathology review, not the grade at the time of enrollment and cohort assignment. The previous publication of this study, including its appendix materials^6^, provides a detailed rationale and methodology for this process. In cohort 1, 50% of patient pathology specimens were classified as low grade and 50% as intermediate grade by the enrolling institution, and many were up-graded on central review: 15% remained low grade, 59% were intermediate, and 26% were high grade. In cohort 2, 100% were classified as high grades by the institution, and on re-classification, 2% were actually low grades, 24% were intermediate, and 74% remained high grades. Median follow-up from definitive surgery on the 239 patients still being followed was 19.2 years (interquartile range 17.3-20.5 years), with 205/239 (86%) followed for at least 15 years, and 87/239 (36%) followed for at least 20 years.

**Table 1:** Patient, DCIS, and treatment characteristics

	Cohort 1 *n* = 561	Cohort 2 *n* = 104
Age	60	58.5
Median	(IQ range 51,70)	(IQ range 50,68)
*Age*		
28–39	13 (2%)	4 (4%)
40–49	93 (17%)	21 (20%)
50–59	166 (30%)	30 (29%)
60–88	289 (52%)	49 (47%)
*Menopause*		
Pre	134 (24%)	29 (28%)
Post		75 (72%)
*Race/ethnicity*		
White	519 (93%)	95 (95%)
Hispanic	8 (1%)	1 (1%)
Black	16 (3%)	4 (4%)
Other	14 (2%)	0 (0%)
Unknown	4	4
*Min margin width*		
<1 mm	9 (2%)	2 (2%)
1.0–2.9 mm	10 (2%)	2 (2%)
3.0–4.9 mm	184 (33%)	28 (27%)
5.0–9.9 mm	239 (43%)	47 (45%)
≥10.0 mm	119 (21%)	25 (24%)
DCIS size		
≤5 mm	226 (40%)	28 (27%)
6–10 mm	231 (41%)	61 (59%)
>10 mm	104 (19%)	15 (14%)
Median size	6 mm (IQ range 4–9 mm)	7 mm (IQ range 5–9 mm)
Mean size	7.5 mm (SD 4.2 mm)	7.8 mm (SD 5.1 mm)
*Method of detection*		
Microcalcifications	399 (71%)	88 (85%)
Density or mass	93 (17%)	4 (4%)
Both	40 (7%)	7 (7%)
Incidental finding	19 (3%)	5 (5%)
Other	8 (1%)	0 (0%)
Unknown	2	0
*Bloody nipple discharge*		
No	541 (98%)	102 (99%)
Yes	12 (2%)	1 (1%)
Unknown	8	1
*Tamoxifen use before entry*		
No	492 (88%)	93 (89%)
Yes	66 (12%)	11 (11%)
Unknown	3	0
*Prior hormone replacement therapy*		
No	315 (57%)	57 (55%)
Yes	239 (43%)	47 (45%)
Unknown	7	0
*Institutionally defined grade*		
Low/Intermediate	561 (100%)	0 (0%)
High	0 (0%)	104 (100%
CAP Grade^a^		
Low	61(15%)	2(2%)
intermediate	249(59%)	19(24%)
High	110(26%)	59(74%)
Unknown	141	24
Comedo necrosis		
Present	123(29%)	56(70%)
Absent	297(71%)	24(30%)
Unknown	141	24
Follow-up status	561	104
IBE	79 (14.1%)	25 (24%)
Died without IBE	131 (23.4%)	23 (22.1%)
Ipsilateral mastectomy	10 (1.8%)	2 (1.9%)
Chemotherapy prior to IBE^b^	9 (1.6%)	4 (3.8%)
Withdrew consent	43 (7.7%)	7 (6.7%)
Lost to Follow-up	79 (14.1%)	14 (13.5%)
Cause of death: breast cancer	3 (0.5%)	1 (1%)
Other cancer	26 (4.6%)	4 (3.8%)
Other	70 (12.5%)	14 (13.5%)
Unknown	67 (11.9%)	10 (9.6%)
Being followed at time of current analysis	210 (37.4%)	29 (27.9%)

*DCIS* ductal carcinoma in situ, *IQ* interquartile, *SD* standard deviation, *IBE* ipsilateral breast event, *CAP* College of American Pathologists.

^a^CAP grade based on central pathology review as reported in 12-year results^1^.

^b^Patients were censored at the start of chemotherapy; 8 for contralateral breast cancer, and 5 for other cancers.

### Breast cancer events

**Fig. 1:**
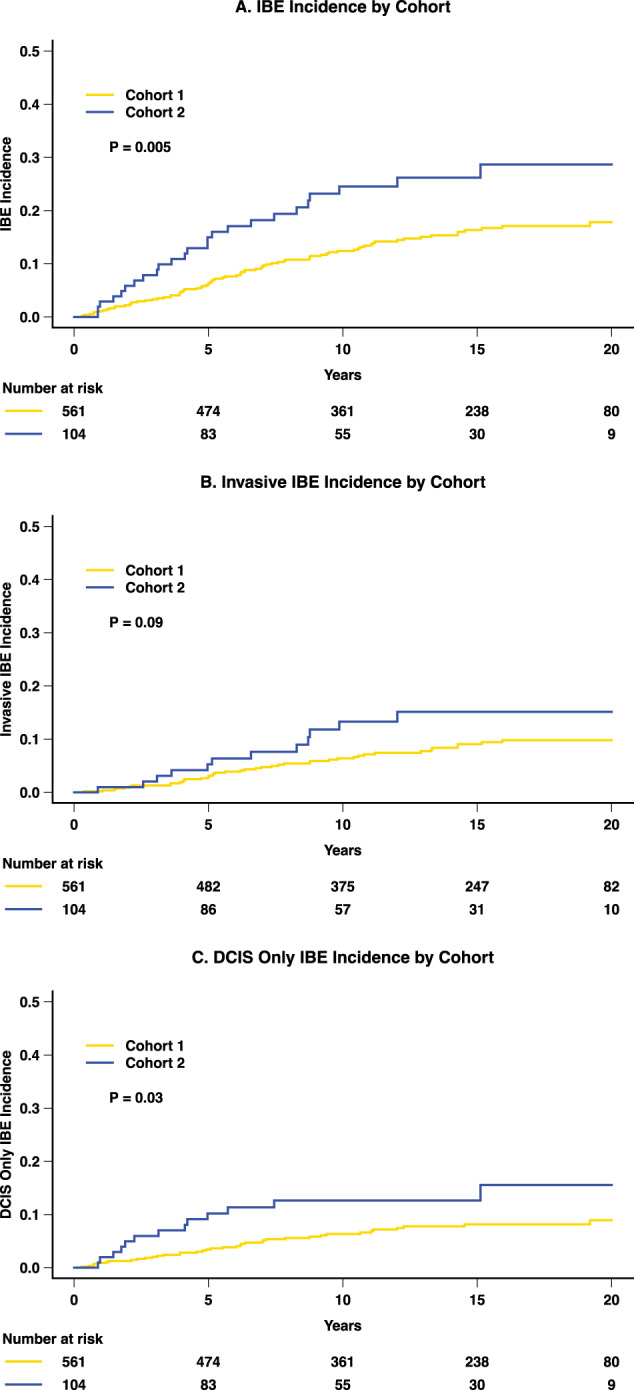
Cumulative incidence of breast events over time. **A** Ipsilateral breast event incidence by cohort. **B** Invasive ipsilateral breast event incidence by cohort. **C** DCIS ipsilateral breast event incidence by cohort.

Overall, there were 104 IBEs (15.5%), 79 in cohort 1 and 25 in cohort 2, of which 54 (51%) were invasive (42 in cohort 1; 12 in cohort 2). The 20-year rates of IBE were 17.8% (95% CI 14.0%, 21.6%) for cohort 1 and 28.7% (95% CI 18.6%, 38.8%) for cohort 2 (*p* = 0.005; Fig. 1, Table 2). The 20-year invasive IBE rates were 9.8% (95% CI 6.9, 12.7) and 15.1% (95% CI 7.0, 23.3), respectively (*p* = 0.09; Fig. 1, Table 2). The rates of IBE and invasive IBE increased over time through 15 years of follow-up and then plateaued. Table 2 summarizes the 10, 15, and 20-year rates of breast events including DCIS, contralateral breast events, and survival. Development of metastatic disease has been reported for 5 patients (0.75%), 4 in cohort 1, and 1 in cohort 2; 2 of these 5 patients have died from breast cancer. In addition, 2 patients have been reported as dying from breast cancer without metastases being reported. In total, 4 patients are reported to have died from breast cancer.

**Table 2:** Breast events and survival

Time	Cohort 1	Annual Rate	Cohort 2	Annual Rate
*Overall IBE rates and 95% confidence intervals*				
10 years	12.4% (9.5%, 15.3%)	1.1%	24.5% (15.7%, 33.4%)	1.75%
15 years	16.4% (12.9%, 19.8%)		26.2% (17.0%, 35.4%)	
20 years	17.8% (14.0%, 21.6%)	0.24%	28.7% (18.6%, 38.8%)	0.5%
*Invasive IBE rates and 95% confidence intervals*				
10 years	6.4 (4.2, 8.6)	0.6%	13.3 (5.9, 20.7)	1%
15 years	9.1 (6.3, 11.8)		15.1 (7.0, 23.3)	
20 years	9.8 (6.9, 12.7)	0.14%	15.1 (7.0, 23.3)	0%
*DCIS Only IBE rates and 95% confidence intervals*				
10 years	6.3 (4.2, 8.5)	0.54%	12.6 (5.9, 19.4)	0.84%
15 years	8.1 (5.6, 10.7)		12.6 (5.9, 19.4)	
20 years	8.9 (6.0, 11.9)	0.16%	15.6 (7.0, 24.1)	0.6%
*Contralateral breast event rates and 95% confidence intervals*				
10 years	5.5 (3.5, 7.5)	0.52%	10.4 (3.9, 16.9)	0.79%
15 years	7.8 (5.3, 10.3)		11.9 (4.8, 18.9)	
20 years	7.8 (5.3, 10.3)	0%	11.9 (4.8, 18.9)	0%
*OS rates and 95% confidence intervals*				
10 years	88.5% (85.8%, 91.2%)		85.7% (78.7%, 92.6%)	
15 years	75.6% (71.7%, 79.4%)		78.7% (70.4%, 87.1%)	
20 years	64.7% (60.1%, 69.3%)		64.8% (53.8%, 75.7%)	

*IBE* ipsilateral breast event, *DCIS* ductal carcinoma in situ, *OS* overall survival.

### Predictors of breast cancer events

Table 3 presents the IBE rate in the overall cohort by age, margin width, size, and CAP grade. Multi-factor Cox proportional hazards model analysis of IBE incidence tables is shown in Supplement 1. Cohort (1 vs. 2 hazard ratio [HR] = 1.77, 95% CI = 1.12, 2.78, *p* = 0.01) and increasing DCIS size (6–10 mm vs. ≤5 HR = 1.51, 95% CI 0.95, 2.40, >10 vs. ≤5 HR = 2.20, 95% CI 1.29, 3.75, *p* = 0.01) were significantly associated with IBE (Supplemental Table 1). Several multivariable models were prepared to evaluate the impact of CAP grade on IBE (Supplemental Table 2). When CAP grade was the only variable in the model, the estimated hazard ratio for CAP high grade vs. CAP low or intermediate grade was 1.56 (95% CI 1.01, 2.41), *p* = 0.04, and was 1.27 (95% CI 0.79, 2.05), *p* = 0.32 in the model with Cohort and tumor size. Finally, when CAP grade was analyzed in a model with size as the only other factor, the estimated hazard ratio was 1.5 (95% CI 0.97, 2.32), *p* = 0.07.

**Table 3:** IBE Rate in overall cohort by selected risk factors

Group	*n*	20-year IBE rate (%)	95% Confidence interval
Age (years)			
≤49	131	24.4	15.8–33.0
50–69	370	18.3	14.0–22.7
≥70	164	15.4	8.9– 21.9
Minimum margin width (mm)			
<5	235	19.0	13.3–24.7
5–9	286	19.3	13.5–25.2
≥10	144	20.7	13.3–28.2
Tumor size (mm)			
≤5	254	13.1	8.6–17.7
6–10	292	21.8	16.1–27.5
>10	119	30.0	18.1–41.8
Grade (CAP guidelines)			
Low	63	15.5	5.3–25.6
Intermediate	268	17.6	12.4–22.9
High	169	26.7	18.5–34.9

*IBE* ipsilateral breast event, *mm* millimeters, *CAP* College of American Pathologists.

Two hundred patients, 175 (31%) in cohort 1 and 25 (24%) in cohort 2, received tamoxifen treatment during the study period. Tamoxifen use was also examined as a time-dependent covariate in proportional hazard models. The estimated hazard ratio for tamoxifen use vs. no tamoxifen use was 0.73 (95% CI 0.46, 1.14) *p* = 0.16, when tamoxifen use was the only variable in the model, and was 0.80 (95% CI 0.51, 1.27), *p* = 0.35 in the model with Cohort and tumor size (Table S3).

When added to the model with size and cohort, age (*p* = 0.34), menopause status (*p* = 0.24), margin width (*p* = 0.76), method of detection (*p* = 0.76), prior tamoxifen use (*p* = 0.23), and prior hormone replacement (*p* = 0.74) was not significant (Supplemental Tables 3–9). None of the factors considered for association with IBE risk showed significant association with invasive IBE risk. No differences were seen between the two cohorts for the 20-year rates of survival [64.7% (95% CI 60.1%, 69.3%) vs. 64.8% (95% CI 53.8%, 75.7%), *p* = 0.60].

## Discussion

Prior reports of this study have demonstrated that for patients with DCIS who were selected on the basis of clinical/pathologic characteristics and treated with lumpectomy without radiation, the rates of developing an IBE and invasive IBE increased over time through 12 years of follow-up. The current report demonstrates that while IBE rates continue to increase over time, the rate is reduced after about 15 years, with minimal increases beyond that time in both cohorts of patients. Though the number of evaluable patients declined with longer follow-up, confidence intervals between 15 and 20 years remained relatively constant and at a statistically significant value. Multiple other studies also demonstrate an increase in rate over time up to 15 years^3,5,11–13^, yet few studies report a recurrence rate beyond this timeframe. The randomized SweDCIS study reported a 20-year IBE rate of 20.0% in patients receiving RT and 32.0% in those treated without RT, though the eligibility criteria for this study were much broader, requiring only that DCIS occupied a quadrant or less of the breast, without specific grade, margin, or size criteria. The hazard ratio for IBE decreased after 12 years in both arms and was close to zero for new in-situ events and remained low for invasive events; the authors speculated that RT delayed rather than prevented invasive IBE^13^. The finding in our analysis that the IBE rate decreases after about 15 years is thus consistent with other analyses and suggests that in a selected low-risk population of mostly postmenopausal patients, the rates of both DCIS-IBE and invasive IBE may level off over time. It is possible that this leveling-off relates to decreased incidence of new breast cancers and /or lower utilization of screening mammography in older patients, though follow-up in this dataset required an annual mammogram. Regardless of these potential explanations, the finding that the event rate levels off after 15 years is useful information for counseling patients.

Importantly, the current analysis continues to show a significant difference in IBE rate between the two cohorts. While the two cohorts were originally specified to include low/intermediate-grade DCIS up to 2.5 cm and smaller high-grade DCIS up to 1 cm, grade re-classification based on updated CAP standards resulted in significant changes, as previously described^6,10^. After central pathology review, 26% in cohort 1 were centrally recategorized as high-grade and 26% in cohort 2 as low- or intermediate-grade Thus, grade reclassification resulted in up-grading many patients’ pathology in cohort 1 and down-grading in cohort 2^6^, highlighting the challenges of evaluating the impact of DCIS grade in longitudinal datasets. On the current univariable analysis evaluating CAP grade alone, CAP grade was significantly associated with 20-year IBE; however, significance was lost on multivariable analyses including size and cohort. Crude IBE rates were higher in patients with higher-grade pathology. Due to the changes in grade classification and the strong relationship between the cohort and the CAP pathology grade, it is difficult to draw strong conclusions regarding the specific impact of grade on IBE outcomes in this study, though grade is classically considered a risk factor for recurrence in most datasets^5,14,15^. On the other hand, though size eligibility requirements differed between the two cohorts, the mean DCIS size was similar in both cohorts, and size was the only factor other than the cohort that was significantly associated with increased IBE rates.

Thus, the underlying difference between the two cohorts is not entirely clear and highlights the fact that predictors of IBE are not fully understood. More recent efforts have focused on using molecular markers to determine the risk of recurrence after surgical excision, including the Oncotype DCIS Score (EXACT Sciences, Madison, WI)^7,16^ and the DCISionRT (PreludeDx, Laguna Hills, CA)^8,9,17–19^. The Oncotype DCIS Score was validated using banked tissue from E5194 and found significantly lower 10-year IBR rates in the low-risk group (IBR 10.6%) versus the intermediate-risk (IBR 26.7%) and high-risk (IBR 25.9%) groups^7^, but the results were not reported by the original cohort to which patients were assigned. The value of molecular markers compared with traditional clinical/pathologic features of DCIS therefore remains an area of great interest. The available data regarding these assays suggest that they may identify the risk of recurrence beyond routinely available clinicopathologic features, pointing to a need to prospectively evaluate these tests in randomized trials.

Both RTOG 9804 and E5194 allowed for the use of tamoxifen but did not specify criteria for its use. In E5194, tamoxifen was prescribed to about 30% of patients, and in RTOG 9804 58% of patients received RT versus 66% of patients not receiving RT. Tamoxifen did reduce the risk of IBE in RTOG 9804 but was not significantly associated with IBE in E5194. The relative impact of tamoxifen, or endocrine therapy in general, in reducing invasive IBE, non-invasive IBE, and contralateral breast events, and its relationship to other risk factors such as DCIS size and grade, is another area of great interest that should be evaluated in future studies. RTOG 9804 and E5914 both required margin widths of 3 mm or greater, though modern margin guidelines require only 2 mm^20^. Margin width beyond 3 mm was not significantly associated with IBE in either study, and the impact of smaller margins cannot be assessed here.

## Methods

### Patient methods

The ECOG-ACRIN E5194 study (NCT00002934) was a prospective, nonrandomized clinical trial that accrued from 1997-2002. The study protocol was approved by the National Cancer Institute Cancer Therapeutic Evaluation Program (NCI/CTEP) and then by the IRB of the respective institutional review boards of the participating centers at which the patients were enrolled. All patients gave written informed consent. Study teams complied with all relevant ethical regulations including the Declaration of Helsinki. Detailed information on trial design and conduct was previously reported^6^. Briefly, the study included two patient cohorts (not randomly assigned) with what was felt at the time of study design to represent low-risk clinical/pathologic characteristics: cohort 1: low- or intermediate-grade DCIS ≤ 2.5 cm (561 patients); and cohort 2: high-grade DCIS ≤ 1 cm (104 patients). As described in the introduction and previously reported, grade at the time of enrollment was determined by the enrolling site, based on standard criteria at that time^21^. Since the original trial design, guidelines for determining DCIS grade changed^10^. Central pathology review was therefore carried out on 500 of the enrolled patients (75% of all patients) after all enrollment had been completed and classifications were updated for subsequent analysis and reported as “College of American Pathologists (CAP) grade”^6^. Margin width was specified to be ≥3 mm in all dimensions, and 96% of pathology specimens met this criterion. All patients underwent WLE without RT. Tamoxifen was prescribed in 30% of all patients. Patients were followed with annual mammograms and breast examinations as specified in the study protocol.

### Statistical methods

The primary endpoint of the study was the rate of IBE (defined as the occurrence of invasive cancer of any histology or DCIS in the treated breast). Time to first IBE was measured from primary surgery to diagnosis of invasive or non-invasive recurrence in the ipsilateral breast. Follow-up for IBEs was censored at the time of an ipsilateral mastectomy, at the time of initiation of chemotherapy for recurrent breast cancer, or at the time of the last disease evaluation. Statistical methods have been previously reported^6^ and are summarized here. The Kaplan–Meier method was used to estimate time-to-event distributions. Confidence intervals (CIs) were constructed using the normal approximation on the probability scale and are given at the 95% level. Cox proportional hazard methods were used to estimate hazard ratios (HRs) and tests for significance for event times. All p-values are two-sided. The multi-variable analysis of risk factors for IBE used the central pathology assignment, rather than the assigned grade at enrollment, and is referred to as the CAP grade. In conclusion: For selected lower-risk patients with DCIS treated with excision without radiation, the rate of IBE increased with size and assigned cohort through 15 years but not beyond. Further studies are needed to refine risk stratification for IBEs after a diagnosis of DCIS, including the contribution of genomic assays on clinical decision-making regarding the use of RT.

### Reporting summary

Further information on research design is available in the Nature Research Reporting Summary linked to this article.

### Supplementary information

Supplemental File

reporting summary

## Data Availability

The study data used for this manuscript can be obtained by submitting a request to the ECOG-ACRIN Cancer Research Group. A request may be initiated by submitting an inquiry through the Contact Us link at https://ecog-acrin.org.
