# Supplementary material for: Lumpectomy without radiation for ductal carcinoma in situ of the breast: 20-year results from the ECOG-ACRIN E5194 study
Source: NPJ Breast Cancer. 2024 Feb 24;10:16. doi: 10.1038/s41523-024-00622-w (PMC10891055; doi:10.1038/s41523-024-00622-w)
Supplement: Supplementary file 1 — Supplemental File [file 41523_2024_622_MOESM1_ESM.pdf]

Supplemental File 1: Estimated Hazard Ratios, 95% Confidence Intervals, and P-values in Proportional Hazards Models for IBE Rate

The following table gives results for the primary model with the two most significant factors.

Supplemental Table 1. Model for Cohort (1, 2) and Lesion Size (<=5 mm, 6-10 mm, >10 mm); n=665

Effect	Est Ratio	Conf Int	p-value
Cohort 2 vs. 1	1.77	(1.12, 2.78)	0.01
Lesion Size 6-10 vs. <=5	1.51	(0.95, 2.40)	0.01
Lesion Size >10 vs. <=5	2.20	(1.28, 3.75)	

Other factors were assessed individually and by separately adding each to the model above. Each table below gives results for two models, the first for the factor alone, the second for the factor in the model with Cohort and Lesion Size (modeled as above). For factors with more than 2 levels, p-values are from Wald tests of no difference among all levels. Patients with missing values for any covariates in a particular model are omitted from that model.

Due to association between Cohort and CAP Grade, the models with just Lesion Size and CAP Grade were also considered in the following table:

Supplemental Table 2. Estimated Hazard Ratios and 95% Confidence Intervals for Models for CAP Grade (Low/Intermed., High); n=500

Effect	CAP Grade alone (CAP Grade p=0.04)	CAP Grade, Cohort, Lesion Size (CAP Grade p=0.32, Cohort P=0.05, Lesion Size p=0.13)	CAP Grade, Lesion Size (CAP Grade p=0.07, Lesion Size p=0.12)
Cohort 2 vs. 1	--	1.72 (0.99, 2.96)	--
Lesion Size 6-10 vs. <=5	--	1.23 (0.73, 2.06)	1.29 (0.77, 2.15)
Lesion Size >10 vs. <=5	--	1.81 (1.01, 3.27)	1.86 (1.03, 3.35)
CAP Grade High vs. Low/Int.	1.56 (1.01, 2.41)	1.27 (0.79, 2.05)	1.50 (0.97, 2.32)

Supplemental Table 3. Estimated Hazard Ratios and 95% Confidence Intervals for Models including Tamoxifen use modeled as a time dependent covariate (the Tamoxifen use variable is 0 until Tamoxifen is started, then has the value 1 after Tamoxifen is started. n=665

Effect	Tam use alone (Tam use p=0.16)	Tam use, Cohort, Lesion Size (Tam use p=0.35, Cohort P=0.02, Lesion Size p=0.02)
Cohort 2 vs. 1	--	1.73 (1.10, 2.74)
Lesion Size 6-10 vs. <=5	--	1.49 (0.94, 2.74)
Lesion Size >10 vs. <=5	--	2.16 (1.26, 3.67)
Tamoxifen use	0.73 (0.46, 1.14)	0.80 (0.51, 1.27)

The following tables give results for other factors considered. The estimated Cohort and Lesion Size effects vary somewhat depending on the other factors in the model, but not very substantially and are omitted.

Supplemental Table 4. Estimated Hazard Ratios and 95% Confidence Intervals for Age (28-39, 40-49, 50-59, 60-88); n=665

Effect	Age alone (Age p=0.40)	Age, Cohort, Lesion Size (Age p=0.34)
Age 40-49 vs. 28-39	0.61 (0.23, 1.61)	0.61 (0.23, 1.61)
Age 50-59 vs. 28-39	0.50 (0.19, 1.28)	0.49 (0.19, 1.27)
Age 60-88 vs. 28-39	0.49 (0.20, 1.24)	0.46 (0.18, 1.17)

Supplemental Table 5. Estimated Hazard Ratios and 95% Confidence Intervals for Menopause Status (Pre, Post); n=665

Effect	Menopause Status alone (Menopause status p=0.29)	Menopause status, Cohort, Lesion Size (Menopause status p=0.24)
Post vs. Pre	0.80 (0.53, 1.21)	0.78 (0.51, 1.18)

Supplemental Table 6. Estimated Hazard Ratios and 95% Confidence Intervals for Margin Width (< 1 mm, 1.0-2.9 mm, 3.0–4.9 mm, 5.0–9.9 mm, >=10.0 mm); n=665

Effect	Margin Width alone (Margin Width p=0.79)	Margin Width, Cohort, Lesion Size (Margin Width p=0.76)
1.0-2.9 vs. <1 mm	1.99 (0.18,21.98)	2.81 (0.25,31.09)
3.0-4.9 vs. <1 mm	1.88 (0.26,13.72)	3.12 (0.42,23.28)
5.0-9.9 vs. <1 mm	1.72 (0.24,12.46)	2.62 (0.35,19.38)
>= 10.0 vs. <1 mm	2.01 (0.27,14.81)	3.16 (0.42,23.80)

Supplemental Table 7. Estimated Hazard Ratios and 95% Confidence Intervals for Method of Detection (MOD) (Microcalcifications, Density or mass, Both, Incidental finding, Other); n=663

Effect	MOD alone (MOD p=0.70)	MOD, Cohort, Lesion Size (MOD p=0.76)
Density or mass vs. Microcalcifications	0.77 (0.42, 1.42)	0.77 (0.42, 1.43)
Both vs. Microcalcifications	1.19 (0.60, 2.38)	1.14 (0.57, 2.27)
Incidental finding vs. Microcalcifications	0.47 (0.12, 1.93)	0.50 (0.12, 2.02)
Other vs. Microcalcifications	0.72 (0.10, 5.17)	0.79 (0.11, 5.73)

Supplemental Table 8. Estimated Hazard Ratios and 95% Confidence Intervals for Prior Tamoxifen Use (pTam) (Yes, No); n=660

Effect	pTam alone (pTam p=0.21)	pTam, Cohort, Lesion Size (pTam p=0.23)
Yes vs. No	0.63 (0.31, 1.30)	0.64 (0.31, 1.32)

Supplemental Table 9. Estimated Hazard Ratios and 95% Confidence Intervals for Prior Hormone Replacement Therapy (pHRT) (Yes, No); n=658

Effect	pHRT alone (pHRT p=0.67)	pHRT, Cohort, Lesion Size (pHRT p=0.74)
Yes vs. No	0.92 (0.62, 1.36)	0.94 (0.63, 1.39)
