# Supplementary material for: Lumpectomy without radiation for ductal carcinoma in situ of the breast: 20-year results from the ECOG-ACRIN E5194 study
Source: NPJ Breast Cancer. 2024 Feb 24;10:16. doi: 10.1038/s41523-024-00622-w (PMC10891055; doi:10.1038/s41523-024-00622-w)
Supplement: Supplementary file 2 — reporting summary [file 41523_2024_622_MOESM2_ESM.pdf]

nature portfolio

Corresponding author(s): Jean L Wright MD

Last updated by author(s): Dec 18, 2023

Reporting Summary

Nature Portfolio wishes to improve the reproducibility of the work that we publish. This form provides structure for consistency and transparency in reporting. For further information on Nature Portfolio policies, see our Editorial Policies and the Editorial Policy Checklist.

Statistics

For all statistical analyses, confirm that the following items are present in the figure legend, table legend, main text, or Methods section.

n/a	Confirmed	
☐	☒	The exact sample size (*n*) for each experimental group/condition, given as a discrete number and unit of measurement
☒	☐	A statement on whether measurements were taken from distinct samples or whether the same sample was measured repeatedly
☐	☒	The statistical test(s) used AND whether they are one- or two-sided<br>*Only common tests should be described solely by name; describe more complex techniques in the Methods section.*
☐	☒	A description of all covariates tested
☐	☒	A description of any assumptions or corrections, such as tests of normality and adjustment for multiple comparisons
☐	☒	A full description of the statistical parameters including central tendency (e.g. means) or other basic estimates (e.g. regression coefficient) AND variation (e.g. standard deviation) or associated estimates of uncertainty (e.g. confidence intervals)
☐	☒	For null hypothesis testing, the test statistic (e.g. *F*, *t*, *r*) with confidence intervals, effect sizes, degrees of freedom and *P* value noted<br>*Give P values as exact values whenever suitable.*
☒	☐	For Bayesian analysis, information on the choice of priors and Markov chain Monte Carlo settings
☒	☐	For hierarchical and complex designs, identification of the appropriate level for tests and full reporting of outcomes
☒	☐	Estimates of effect sizes (e.g. Cohen's *d*, Pearson's *r*), indicating how they were calculated

Our web collection on statistics for biologists contains articles on many of the points above.

Software and code

Policy information about availability of computer code

Data collection	N/A
Data analysis	All analyses were performed in R version 4.2.1

For manuscripts utilizing custom algorithms or software that are central to the research but not yet described in published literature, software must be made available to editors and reviewers. We strongly encourage code deposition in a community repository (e.g. GitHub). See the Nature Portfolio guidelines for submitting code & software for further information.

Data

Policy information about availability of data

All manuscripts must include a data availability statement. This statement should provide the following information, where applicable:

- Accession codes, unique identifiers, or web links for publicly available datasets
- A description of any restrictions on data availability
- For clinical datasets or third party data, please ensure that the statement adheres to our policy

The data from the present publication will be made available by request from the NCTN/NCORP Data Archive: https://nctn-data-archive.nci.nih.gov

Research involving human participants, their data, or biological material

Policy information about studies with human participants or human data. See also policy information about sex, gender (identity/presentation), and sexual orientation and race, ethnicity and racism.

Reporting on sex and gender	The eligibility for participation in the study are available in the original protocol (NCT00002934) and included female patients.
Reporting on race, ethnicity, or other socially relevant groupings	Patients of all races and ethnicities were included and patient enrollment by race and ethnicity is reported here, based on categorization at the time of enrollment.
Population characteristics	The eligibility for participation in the study required patients to be age 18 or older. Age is reported in the manuscript and tables including range, median, and interquartile range.
Recruitment	Recruitment was carried out according to standard procedures at the time of study accrual in sites that were eligible to enroll in ECOG-ACRIN studies.
Ethics oversight	The study protocol was approved by the respective institutional review boards of the participating centers at which patients were enrolled. All patients gave written consent. Study oversight was carried out by the ECOG-ACRIN Data Safey and Monitoring Board.

Note that full information on the approval of the study protocol must also be provided in the manuscript.

Field-specific reporting

Please select the one below that is the best fit for your research. If you are not sure, read the appropriate sections before making your selection.

☒ Life sciences ☐ Behavioural & social sciences ☐ Ecological, evolutionary & environmental sciences

For a reference copy of the document with all sections, see nature.com/documents/nr-reporting-summary-flat.pdf

Life sciences study design

All studies must disclose on these points even when the disclosure is negative.

Sample size	665 (561 in Cohort 1, 104 in Cohort 2)
Data exclusions	Inclusion and exclusion criteria are reported in the study protocol.
Replication	Not applicable
Randomization	Not a randomized study
Blinding	Not a blinded study

Reporting for specific materials, systems and methods

We require information from authors about some types of materials, experimental systems and methods used in many studies. Here, indicate whether each material, system or method listed is relevant to your study. If you are not sure if a list item applies to your research, read the appropriate section before selecting a response.

Materials & experimental systems

n/a	Involved in the study	
☒	☐	Antibodies
☒	☐	Eukaryotic cell lines
☒	☐	Palaeontology and archaeology
☒	☐	Animals and other organisms
☐	☒	Clinical data
☒	☐	Dual use research of concern
☒	☐	Plants

Methods

n/a	Involved in the study	
☒	☐	ChIP-seq
☒	☐	Flow cytometry
☒	☐	MRI-based neuroimaging

Clinical data

Policy information about clinical studies

All manuscripts should comply with the ICMJE guidelines for publication of clinical research and a completed CONSORT checklist must be included with all submissions.

Clinical trial registration	NCT00002934
Study protocol	CT.Gov
Data collection	Patients were accrued and data collected at participating ECOG-ACRIN sites between 1997-2002.
Outcomes	Primary and secondary outcome measures were pre-specified in the orginal protocol. Primary outcome was ipsilateral breast event (IBE) defined as occurrence of invasive cancer of any histology or DCIS in the treated breast. This is stated in the manuscript.

Plants

Seed stocks	N/A
Novel plant genotypes	N/A
Authentication	N/A
